# Feeding Infants

**Published:** 1884-08

**Authors:** 


FEEDING INFANTS.—Infants should never be fed from a bottle, but always from a spoon or cup. This is the only way to prevent them from being over-fed, which, with the refusal to them of a free supply of water, that idiots still practice, kills three-fourths of the hand-fed children that die. Water should always be offered before milk is given; otherwise, to quench thirst, and not from hunger, they will drink more of the latter than they can digest, and a bellyful of trouble will ensue.—*N. Y. Medical Journal.*

